# Health Facilities Safety in Natural Disasters: Experiences and Challenges from South East Europe

**DOI:** 10.3390/ijerph9051677

**Published:** 2012-05-04

**Authors:** Vesela Radovic, Ksenija Vitale, Paul B. Tchounwou

**Affiliations:** 1 The Faculty of Environmental Protection, University EDUCONS, 85 Vojvode Putnika, 21208 Sremska Kamenica, AP Vojvodina, Serbia; Email: veselaradovic@yahoo.com; 2 Medical School, School of Public Health, University of Zagreb, 4 Rockefeller Street, 10000 Zagreb, Croatia; 3 Molecular Toxicology Research Laboratory, NIH-Center for Environmental Health, College of Science, Engineering and Technology, Jackson State University, 1400 Lynch Street, P.O. Box 18540, Jackson, MS 39217, USA; Email: paul.b.tchounwou@jsums.edu

**Keywords:** environmental security, natural disasters, safety, hospitals planning, critical infrastructure

## Abstract

The United Nations named 2010 as a year of natural disasters, and launched a worldwide campaign to improve the safety of schools and hospitals from natural disasters. In the region of South East Europe, Croatia and Serbia have suffered the greatest impacts of natural disasters on their communities and health facilities. In this paper the disaster management approaches of the two countries are compared, with a special emphasis on the existing technological and legislative systems for safety and protection of health facilities and people. Strategic measures that should be taken in future to provide better safety for health facilities and populations, based on the best practices and positive experiences in other countries are recommended. Due to the expected consequences of global climate change in the region and the increased different environmental risks both countries need to refine their disaster preparedness strategies. Also, in the South East Europe, the effects of a natural disaster are amplified in the health sector due to its critical medical infrastructure. Therefore, the principles of environmental security should be implemented in public health policies in the described region, along with principles of disaster management through regional collaborations.

## 1. Introduction

The global impact of natural disasters is greater than ever before. In 2010 worldwide there were 385 natural disasters, of which 79% were meteorological, killing 297,000 people, affecting over 217 million, and causing $123.9 billion in economic damage [1]. The question is how to return to normal life and mitigate disaster consequences at the same time, particularly in the health care sector and hospitals as a critical infrastructure.

In the international policy and the academic community there were many actions taken in the search for solutions for improving preparedness and responses to humanitarian emergencies, while at the same time providing continuation of work. The World Conference on Disaster Reduction, held in Japan 2005, established the Hyogo Framework for Action 2005–2015: Building the Resilience of Nations and Communities to Disasters [2]. The conclusions and recommendations were focused on how to: (1) reduce the underlying risk factors and perform social and economic development practices with integrated disaster risk reduction planning into the health sector; (2) promote the goal of “hospitals safe from disaster” by ensuring that all new hospitals are built with a level of resilience that strengthens their capacity to remain functional in disaster situations; and (3) implement mitigation measures to reinforce existing health facilities, particularly those providing primary health care [3].

Every two years, the United Nations International Strategy for Disaster Reduction Secretariat (UNISDR) selects a topic that reflects one of the five priorities of the Hyogo Framework for Action 2005–2015. The theme of the 2008–2009 world campaign was: Hospitals Safe from Disasters: Reduce Risk, Protect Health Facilities, and Save Lives. The UNISDR coordinated campaign in partnership with the World Health Organization (WHO). The efforts were supported from the Global Facility for Disaster Reduction and Recovery of the World Bank, in partnership with governments, international and regional organizations, non-governmental organizations, trying to raise awareness about why and how to protect health facilities and ensure their functioning during and in the aftermath of disasters. This campaign addresses the topic: “Hospitals Safe from Disasters” in line with the UNISDR’s mandated focus on natural hazards without considering other safety issues. Some of the European countries joined the campaign, Serbia and Croatia among them. Both countries belong to the region of “South East Europe with a history of vulnerability to disasters caused by natural and technological hazards, many of which transcend borders and exceed the management capacities of individual countries” [4]. During the last few years both countries suffered considerable economic losses caused by the weather related natural disasters. That is a reason to develop policies and management strategies that would be more regionally oriented with a common goal of sharing experiences and resources. The aim of this article is to present principles of crisis management of the health facilities safety in Croatia and Serbia within the European Union (EU) context, and explore possibilities of disaster management through regional collaboration.

## 2. Organizational Response to Disasters in Europe, Serbia and Croatia

Europe is experiencing an increasing number of disasters due to natural hazards. Disaster risk reduction and management in EU has shifted from a response-oriented approach towards an Integrated Risk Management (IRM) approach that includes prevention, preparedness, response and recovery [5]. Over the years, the EU has developed major tools through which all its policy objectives in the field of civil protection may be achieved. The Community Action Program, which supported projects in the field of prevention, preparedness and response to disasters caused by natural hazards, was adopted in 1999 and completed in 2006. The Community Civil Protection Mechanism, created in 2001 to reinforce the cooperation in civil protection assistance interventions, has now developed into a robust platform for European Civil Protection Cooperation. Thirty countries (27 EU Members, Iceland, Liechtenstein and Norway; Croatia joined in 2009) participate in the CCP Mechanism, ensuring an effective delivery of assistance in emergencies which may require urgent responses. In September 2010, Slovenia and Croatia experienced unusually heavy rainfall with significant damage to their energy, water and wastewater, transport, education and health infrastructures. The European Commission awarded aid to Slovenia and Croatia. This aid is provided by the Solidarity Fund (SF), which finance the emergency measures taken by national, regional and local authorities, such as rescue operations, cleaning of affected areas and rehabilitation of infrastructure of primary importance. Croatia as accession country, submitted its application, but it was less than the threshold for intervention. However, according to legislation, a country affected by the same major disaster as a neighboring EU country (in this case Slovenia) may exceptionally benefit from the SF. Croatia received almost €4 million in aid from the SF in 2010 that will be used to reimburse the cost of cleaning-up operations of disaster stricken zones [6].

Croatia will become EU member in the near future, being the first new member since 2007. The Republic of Serbia made considerable progress in the process of approaching EU membership but still has obstacles on its path. The European Parliament ratified the Stabilization and Association Agreement between the EU and Serbia in January 2011. Regardless of a different status in approaching EU membership, increasing level of cooperation between the two neighboring countries, mostly in the area of the economic cooperation and security is an encouraging fact.

Croatia and Serbia has shared the same history in the civil protection area as a part of former Republic of Yugoslavia. The well recognized specific model of disaster management, well known in the academic community as “Yugoslav model”, was organized in 1963 as a part of the Ministry of Defense, where Armed Forces, the Civil Protection Service and, Monitoring and Alert service were integrated for the same tasks. The main task of the Civil Protection Service was the protection and rescues of people, material and other assets as well as the mitigation of the effects of the war, natural disasters and other dangers [7]. The concept of humanitarian protection continued to evolve through 20th century beyond its initial focus on the armed conflict, but the period of conflicts during nineties ended the common history of Yugoslav Civil Defense System.

In Serbia in 1995, the Law on People Defense transformed into the Civil Protection Service, expanding its responsibility to include protection and rescue in natural disaster. The Serbian Law on Defense from 1994 established the Territorial Body for Defense which was renamed the Department for Defense of the Republic of Serbia in 2000, but the legal framework for disaster response had been created for a country and a political system that no longer existed. That opened space for other government structures to promote their responsibility for various aspect of crisis response and step into the vacuum which was created [8]. In 2009, the Law on Emergency Situations was passed, along with sub-laws [9]. The Sector for Emergency Management was established, integrating the existing resources within protection, rescuing and reacting in emergency situations, with the aim of strengthening the institutional structure and capacity for preventive actions in the cases of natural disasters. The Sector has several operational units and its functionality is ensured through territorial organization of twenty seven units. It consists of the Department for Prevention; Department for Fire and Rescue Units; Department for Risk Management; Department for Civil Protection; The National Training Centre. This Sector became a full member of the Partial European and Mediterranean Major Hazards Agreement of the Council of Europe (EUROPA) [10]. The existing Law on Emergency Situations still does not have all necessary legal and organizational prerequisites for reaching maximum efficiency. National strategy to decrease the risk and to improve help, protection and rescue in emergency situations, followed by National Action Plan for the implementation of this Strategy is still missing.

In Croatia the situation is more encouraging. The system has been established earlier and there was more intense cooperation within the regional and international community. All legal directives, strategies, laws and sub-laws were adopted and harmonized with EU. Hence, the system has all necessary conditions to be effective. The National Protection and Rescue Directorate in Croatia is an independent, professional and administrative organization, tasked with preparing plans and managing operational forces as well as coordinating the activities of all participants in the protection and rescue system. The basic tasks of the National Protection and Rescue Directorate are stipulated by the Law on Protection and Rescue [11]. The most important tasks are risk and vulnerability assessment, drafting measures aimed at preventing crises and accidents, ensuring measure implementation, and effective emergency management in case of major disasters. The National Protection and Rescue Directorate consist of Directors Cabinet, International Cooperation Department and Internal Affairs Department as well as five sectors: Civil Protection Sector; Fire Fighting Sector; Sector for 112 Emergency Calls dispatching center; Fire Fighting, Protection and Rescue School and, Personnel, Legal and Finance Sector. The functionality of the Rescue Directorate is ensured through its territorial organization. Each county has a County Protection and Rescue Office consisting of prevention, planning and supervision department and the county 112 center. In county offices of the four biggest cities Zagreb, Rijeka, Osijek and Split there are Protection and Rescue departments, while in the county offices on the coast (Zadar, Šibenik, Split and Dubrovnik) there are in addition State intervention operating units [12].

## 3. Organization of Healthcare in Serbia and Croatia

In both countries health care is free for all services and financed through taxes, with facilities being owned by the state and/or local governments. Services are organized through primary, secondary and tertiary care while structure, number, capacity and spatial distribution are established by local needs. Parallel to the state-owned health care system, privately owned institutions operate as free enterprises or as contractors with state health care system.

In Serbia, according to the Network Plan, the total number of state-owned health care institutions is 375, including 167 health centers, 41 pharmacies, 16 institutes at the primary level, five medical centers, 40 general hospitals, 40 specialized hospitals for acute and chronic conditions and for rehabilitation, five clinical-hospital centers, six clinics, 13 specialized institutes, Military Medical Academy, four clinical centers, 27 institutes and bureaus conducting the business on multiple levels, out of which four are institutes and 23 bureaus of public health. The total number of beds laid down by the Network Plan is 38,590. In addition to state-owned, health care services are also provided in privately owned institutions. At the beginning of 2009, over 5,000 forms of privately owned health care services were registered in the Republic of Serbia (health care institutions and private practice), of which seven are health centers, 72 hospitals, 136 polyclinics, about 1,200 medical offices, 2,000 dental offices, 1,400 pharmacies and 200 different laboratories and diagnostic offices [13].

In Croatia according to the National Institute for Public Health, state owned assets comprise 49 health centers, four clinical hospital centers, 10 clinical hospitals, 23 general hospitals, 26 specialized hospitals with expressed beds and seven without, 10 general wards and five out of hospital maternity wards in small towns. There are 22 institutes for public health, five state institutes (transfusion medicine, health protection and safety at work, toxicology, mental health, emergency medicine). In all type of institutions Croatia has 5.4 beds per 1,000 inhabitants (3.6 acute and 1.8 for chronic patients). In Croatia there are 3,379 private practice units which include doctors’ offices, dentists’ offices, laboratories, pharmacies, physical therapy, home care services. Croatia is with 276 doctors per 100,000 people below the average for transition countries and the EU [14].

## 4. Vulnerable Infrastructure

A major problem of a large number of hospitals in both countries is related to their age. In Croatia 30% of hospitals in use today were constructed in the mid-19th century with limited possibilities for structural improvements. Most of the hospitals were built between 1930s and 1960s and until today under constant reconstruction due to the lack of space and the need for modernization. A considerable number of hospitals are of pavilion type (different wards in different buildings) that makes reconstruction even more difficult and expensive [15]. In the late eighties two military hospitals were built and today one is turned into clinical hospital center in Zagreb and the other has become a general hospital in Knin. At the beginning of 21st century a new cycle of hospital construction started, but it is far from fulfilling current needs. Particular problem are hospitals for which construction was started but never completed due to the lack of money and/or political reasons. The university hospital in Zagreb is an excellent example. After more than 30 years of unfinished construction, the building has deteriorated, and it is highly questionable if such an unpreserved construction can meet modern building criteria such as seismic resistance.

The situation is almost the same in Serbia. Until the middle of 19th century there were no civil hospitals in Serbia. The first one was constructed in 1868 in Belgrade and then in other cities. Today some of those hospitals are under protection as cultural heritage but they are still in operation. Most of the existing hospitals were built after the Second World War. A considerable number were built by community investment and probably this is the reason why they are operating, but were never completely finished. Like in Croatia, in Serbia the main health center was never finished (the construction site still deteriorating). The biggest single investment was a military academy hospital still operating as such. Constant reconstruction and renovation of existing capacities is financially exhausting and their quality is uncertain. Serbia received European Bank loan for reconstruction of its four clinical centers and 20 general hospitals. The latest health facility was built in 2010 in Vojvodina, as an Urgent Health Center and it is the most modern hospital in Serbia financed by a local government [13,16].

## 5. Specific Examples of Health Facility Vulnerability in Serbia and Croatia

The Serbian care health system is facing serious problems due to political circumstances and financial constraints. Democratic change brought expectations for a better future of the health system, and the first recovery signs were visible thanks to enormous number of international donations. Serbia belongs to a region with known influence of climate changes with the significant appearance of hydro-meteorological danger [5]. Each meteorological situation, which causes considerable damage, is classified as a weather-related disaster. During the last decade the greatest number of health facilities in Serbia was endangered due to various weather-related disasters causing great economic losses. Floods, rise of underground water, heavy rains affected a number of general hospitals along with the special hospital for lung tuberculosis and lung diseases in Jaša Tomić village and the hospital for neurological diseases and post traumatic conditions in Slankamen. The main problem is their ineffective construction due to the poor planning situating hospitals in flood areas. Most probably at the time of the construction different environmental conditions were present that make today’s reconstructions extremely difficult. According to contemporary laws on floods the whole village is outside of settlements limit defense line, which shows lack of coordination when planning protection of critical infrastructure such as hospitals. As stated before parts of mentioned buildings are under cultural heritage protection and along with fragile construction sometimes reconstructions are almost impossible. An example is the impossibility of air-conditioning system installation at one hospital. Water scarcity during summer is a common problem in Health Center in Čačak owing to the low capacity of the local water supply system. The most dramatic disaster was an earthquake, measuring 5.4 on the Richter and 7.5 on the Mercalli scale that hit central Serbia in 2009, 15 kilometers northwest from the town of Kraljevo. Two people were killed, 120 sought medical assistance, and damage to around 4,000 structures was recorded. Although the Hospital in Kraljevo had been badly damaged (the operating block and polyclinic sustained the most damage), services were provided to the injured and other people in need [17]. The problem is that the hospital is still awaiting reconstruction. Serbia, in the current financial crisis and the greatest budget deficit in history, is not able to provide funds for the rehabilitation of the community after the earthquake. Unfortunately, it is not the first time that Serbia is faced with a lack of funds in mitigation of consequences of natural disasters in communities [18]. For this purpose, Serbia sought help in the form of European Bank loans. The Project is still under appraisal. Reopening the hospital building requires a rigorous and comprehensive process to ensure that the building is safe and meets all regulatory requirements to provide patient care. However this hospital in Kraljevo was never even closed.

In Croatia the situation is much better. There has been only sporadic and non-significant hospital damage during the disasters, mainly because of better prevention and proper maintenance. Some damages from floods and raised level of underground water were recorded at the general hospitals in Varaždin, 2011 [19] and Gospić [20]. Sometimes damages are caused by heavy rains and improper rebuilding activities like in Osijek or poor maintenance like in the Šibenik hospital [21].

## 6. What Activities Should be Taken in Near Future to Protect Health Facilities

Hospitals represent an enormous investment for any country. They are an extremely important part of “critical infrastructure’’, therefore taking steps to make the hospitals safer and more resilient to natural calamities are needed. Implementing hospital safety from disaster impacts involves more than just protecting physical structures. As it is well known, a safe hospital should not collapse in disasters. It has to continue to function and provide medical services to meet the basic health care needs. With contingency plans in place and health workforce and volunteers trained to keep the network operational the challenge can be met. However, health services are not only critical emergency centers, they play a vital role in recovery, social cohesion and economic stability. Hospitals and primary care centers have central role in health driven development, taking key role in surveillance to prevent outbreaks, organize public health campaigns and acting as focal points for community organizations.

Constant training of health workforce and staff from related sectors along with voluntaries not only from national Red Cross but various levels of civil society and NGO groups, to improve the response capacity of health facilities is needed. In both countries employers have an obligation to train an appropriate number of employees for rescue and evacuation in case of emergency. Special regulations like previously mentioned laws regulate the issues of prevention, fire fighting and evacuation of employees. Employers in emergency management sectors and in health facilities are the main, but should not be the only ones in charge. All the community stakeholders should be involved in mitigating processes according to their capacity. 

In Serbia, the lack of scientific and academic programs about disaster management creates a weakness of the system. Serbia also still does not have a methodology for vulnerability assessment, as one of the most important sub-laws, which enable a process of functional vulnerability assessment of health services network and identification of priorities.

Strengthening preparedness for emergencies and disasters, and mandatory preparing, testing, and updating of national, local and hospital disaster plans are needed as constant activities. During all phases in hospital and emergency management, the Safe Hospitals Checklist components should be followed: location, structural safety, non-structural safety, functional safety (protection and rescue plan, preventive measures, proper maintenance, available resources). That is a reason why management should consider specific work positions and demands for a person who should be a check consultant. In Croatia, after the conducted WHO Project there are five trained evaluators for hospital safety index assessment. Those people are vital for ensuring the safety measures of hospitals in the disaster situation, and this activity cannot be just added in some other employees’ list of duties. Croatia already has taken many improvement steps compared with Serbia. In Croatia a workshop on hospital safety index assessment was organized by WHO/Europe and the WHO Country Office, where the project of six Croatian hospitals on hospital safety index assessment was presented [22]. The assessment was conducted using the WHO assessment tool, giving a safety index indicator for each of the six hospitals involved in the pilot project. The results of this pilot project are valuable as they point to areas where investments are needed in these particular hospitals for their safety to be improved and for enabling them to function in an emergency situation. Croatia is organizing education for educators in medical response to major incidents (MRMI) at the international level. It is a practical course for all stakeholders in the disaster mitigation processes based on real data and past experience. One of the visions is continuation of collaboration between European countries, particularly neighboring countries such as Croatia and Slovenia in order to develop more similar operative plans [23].

## 5. Conclusions

Protecting critical health facilities from disasters is possible by including risk reduction in the design and construction of all new health facilities, and by reducing vulnerability in existing health facilities through selecting and retrofitting the most critical ones.

Therefore, it is necessary to strengthen the capacities of the local community to enable fast reactions in the protection of health facilities and to reduce the influence of the consequences in cases of natural disasters. Apart from that, in Serbia it is urgent to start work on the National Action Plan of Adaptation and on the Strategy for response in emergency cases. Croatia is already implementing the WHO recommendations, as a helpful resource to assess the functioning of health systems with the goal of giving support to the Ministry of Health and Social Welfare in identification of strengths, weaknesses and gaps in the health systems’ crisis preparedness. Principles of environmental security should be implemented in public health policies, along with principles of disaster management through regional collaboration in order to diminish expenses and strengthen efficiency.

## References

[1] Guha-Sapir D., Vos F., Below R., Ponserre S. (2011). Annual Disaster Statistical Review 2010: The Numbers and Trends.

[2] United Nation International Strategy for Disaster Reduction (UNISDR) (2010). Hyogo Framework for Action 2005–2015: Building the Resilience of Nations and Communities to Disasters. www.unisdr.org/eng/hfa/docs/HFA-brochure-English.pdf.

[3] United Nation International Strategy for Disaster Reduction (UNISDR) (2010). Hyogo Framework for Action 2005–2015: Building the Resilience of Nations and Communities to Disasters. Final Report of the Word Conference on Disaster Reduction, 2006 (A/CONF.206/6).

[4] The South Eastern Europe Disaster Risk Mitigation and Adaptation Program (2009). The Structure, Role and Mandate of Civil Protection in Disaster Risk Reduction for South Eastern Europe.

[5] European Environmental Agency (2010). Mapping the Impacts of Natural Hazards and Technological Accidents in Europe. An Overview of the Last Decade; Technical Report No. 13/2010.

[6] The European Union Solidarity Fund (2011). Emergency Aid to Member States and EU Accession Countries Affected by Major Natural Disasters. www.ec.europa.eu.interventions_since_2002_may_2011.

[7] (1983). The Forces of National Defense, Strategy of Armed Struggle.

[8] Earl W., Chu E., Dimitrijevic I. (2008). The Civil Protection System in Serbia.

[9] Law on Emergency Situations. Code of the Republic of Serbia.

[10] Information about International Cooperation and Signed Agreements.

[11] Protection and Rescue Act. Code of the Republic of Croatia.

[12] Organization of Emergency Management in Croatia.

[13] Ministry of Health, Republic of Serbia www.zdravlje.gov.rs.

[14] (2010). Croatian Health Service Yearbook 2009.

[15] Vodička M., Juracic D. (1994). Hospitals (in Croatian).

[16] The Clinical Center of Vojvodina www.kcv.rs/en.

[17] UN Office of the Resident Coordinator (2010). Situation Report No. 1—UN in Serbia. www.rs.one.un.org/6/Kraljevo%20situation%20report%20No.%201.doc.

[18] (2005). Technical Report on Damages on the Buildings of the Lung Diseases Hospital “Dr. Vasa Savić” Jaša Tomić Village Caused by the Floods in April and May 2005.

[19] (2011). Nevrijeme izazvalo poplave i probleme u opskrbi vodom (Floods and Water Supply Problems Caused by Storm).

[20] Gospic Flooded Hospital, Cathedral and Police Station. www.dnevnik.hr/vijesti/hrvatska/nevrijeme-u-gospicu-poplavljeno-srediste-grada.html.

[21] Zekic N. (2008). After Reconstruction Operating Room is Leaking.

[22] WHO (2011). Report on Hospital Safety Index Assessment in Croatia.

[23] Medical News (2011). Medical Care of Mass Accidents: Croatia Became International Educational Center.

